# Imaging techniques to diagnose radiation damage

**DOI:** 10.1186/1470-7330-15-S1-O34

**Published:** 2015-10-02

**Authors:** Massimo Bellomi

**Affiliations:** 1 European Institute of Oncology, Milan, Italy

## 

Radiotherapy affects all tissue of the body, but the evidence and the timing of these changes vary from organ to organ. The details of the radiotherapy, including the volume and shape of the area treated, dose, time from completion of therapy, possible effects of other treatment including chemotherapy, and the variability of human response are all factors in the appearance of radiotherapy change.

Different techniques have to be used according to the organ and to the pathology to be investigate: “conventional” imaging is still a cornerstone in main situation, as swallowing fluoroscopy in irradiated neck, Chest X-ray in radiation pneumonitis, Ultrasound in radiation pericarditis, MRI in postradiation bony changes etc.

The new imaging tools able to evaluate tissue perfusion (both by CT or MRI) and cellularity (Diffusion Weighted MRI) can be applied to evaluate vascular damage prior the ischemic changes and especially in differential diagnosis of fibrosis versus recurrent tumor.

When the patient complains symptoms that can be referred to the previous radiotherapy and imaging documents tissue changes that explain symptoms and are likely due to the treatment, the communication to the patient has to be clear, understandable and bluntly: the base of this relationship is grounded on a correct information on benefits and risks given at the time of treatment.

When tissue changes are incidental finding and not related to any symptom, as for example a mild peripheral lung fibrosis after breast irradiation, this raises the issue of how to report the finding. It has to be done without rising concern in the patient, nor instilling doubt that something went wrong with the therapy and a sentence as “due to normal outcome of radiotherapy” could be suggested if agreed with the Radiotherapist.

In conclusion, the use of radiation to treat primary and metastatic tumors results in damage to normal tissue that is often evident on imaging studies. Treatment techniques have continued to evolve dramatically and techniques such as IMRT and proton therapy are specifically designed to decrease dose and injury to tissues surrounding malignant tumors. However some patients treated with definitive radiation therapy over the past several decades continue to survive and present for surveillance. Evaluation of imaging studies in these patients requires an understanding of the expected changes post therapy.

